# Percutaneous Cannulation for Minimally Invasive Heart Valve Surgery: Results from a Multicenter Registry

**DOI:** 10.1093/ejcts/ezaf219

**Published:** 2025-07-02

**Authors:** Jonas Pausch, Jessica Weimann, Miriam Silaschi, Eissa Alaj, Vahid Seidiramool, Markus Kofler, Jörg Kempfert, Hendrik Treede, Ahmed Ghazy, Thilo Noack, Ibrahim Gadelkarim, Sabine Bleiziffer, Julia Götte, Hermann Reichenspurner, Lenard Conradi

**Affiliations:** Department of Cardiovascular Surgery, University Heart and Vascular Center Hamburg, University Medical Center Hamburg-Eppendorf, 20251 Hamburg, Germany; Department of Cardiology, University Heart and Vascular Center Hamburg, University Medical Center Hamburg-Eppendorf, 20251 Hamburg, Germany; Clinic and Polyclinic for Cardiac Surgery, University Hospital Bonn, 53127 Bonn, Germany; Clinic and Polyclinic for Cardiac Surgery, University Hospital Bonn, 53127 Bonn, Germany; Clinic and Polyclinic for Cardiac Surgery, University Hospital Bonn, 53127 Bonn, Germany; Department of Cardiothoracic and Vascular Surgery, German Heart Center Berlin, 13353 Berlin, Germany; Department of Cardiothoracic and Vascular Surgery, German Heart Center Berlin, 13353 Berlin, Germany; German Center for Cardiovascular Research, Partner Site Berlin, 10785 Berlin, Germany; Clinic and Policlinic for Heart- and Vascular Surgery, University Hospital Mainz, 55131 Mainz, Germany; Clinic and Policlinic for Heart- and Vascular Surgery, University Hospital Mainz, 55131 Mainz, Germany; University Clinic of Cardiac Surgery, Leipzig Heart Center, 04289 Leipzig, Germany; University Clinic of Cardiac Surgery, Leipzig Heart Center, 04289 Leipzig, Germany; Clinic for Thoracic and Cardiovascular Surgery, Heart and Diabetes Center North Rhine-Westphalia, Bad Oeynhausen, 32545 Bad Oeynhausen, Germany; Clinic for Thoracic and Cardiovascular Surgery, Heart and Diabetes Center North Rhine-Westphalia, Bad Oeynhausen, 32545 Bad Oeynhausen, Germany; Department of Cardiovascular Surgery, University Heart and Vascular Center Hamburg, University Medical Center Hamburg-Eppendorf, 20251 Hamburg, Germany; Department of Cardiovascular Surgery, University Heart and Vascular Center Hamburg, University Medical Center Hamburg-Eppendorf, 20251 Hamburg, Germany; Department of Cardiac Surgery, University Hospital Cologne, 50937 Köln, Germany

**Keywords:** minimally invasive heart valve surgery, endoscopic heart valve surgery, vascular closure device, vascular access site closure, plug-based VCD, suture-based VCD, groin complications

## Abstract

**Objective:**

To avoid potential groin incision associated complications and further streamline surgery percutaneous femoral cannulation using different vascular closure devices (VCDs) has emerged to establish cardiopulmonary bypass during minimally invasive heart valve surgery (HVS).

**Design:**

The Percutaneous peRipheral cannulatiOn for Minimally InvaSive heart valve surgEry (PROMISE) multicentre registry included patients, receiving percutaneous vascular access site (VAS) closure during minimally invasive HVS. Retrospective analyses were performed to evaluate major and minor VAS-related complications of plug- (group 1) vs suture-based (group 2) systems according to modified Valve Academic Research Consortium (VARC) 3 criteria (ie, retrograde dissection, vascular injury, conversion to surgical cut-down, or vascular intervention).

**Results:**

In total, 755 patients (66.1% (499/755) male; median age 61.9 years) were included and treated using a plug- (*n* = 450) or suture-based (*n* = 305) VCD. Most prevalent comorbidities were hypertension (53.8%; 335/755) and atrial fibrillation (29.4%; 222/755), resulting in a median STS Prom Score of 0.5%. Prevalence of peripheral artery disease was 4.4% (33/450). Immediate hemostasis was significantly higher in the plug-based group (99.8% (445/450) vs 77.7% (237/305); *P* < .001). Accordingly, application of a second VCD (0.0% (0/450) vs 34.8% (106/305); *P* < .001) as well as conversion rates to surgical cut-down (1.3% (6/450) vs 3.9% (12/305); *P* = .04) were significantly lower. Prevalence of VAS-related complications (ie, arterio-venous (AV) fistula (0.2% (1/450) vs 0% (0/305); *P* > .99), pseudoaneurysm (0.4% (2/450) vs 0% (0/305); *P* = .66), or postoperative VAS bleeding (1.6% (7/450) vs 0.7% (2/305); *P* = .26)) was low in both the groups (2.9% (13/450) vs 5.2% (16/305); *P* = .14).

**Conclusions:**

VAS-related complications were favourably low in both the groups. Plug-based VCDs are potentially associated with significantly higher rates of immediate hemostasis and lower incidence for additional VCD or surgical cut-down. Usage of dedicated VCD (plug- and suture-based) for VAS closure after percutaneous cannulation is feasible, safe, and further decreases invasiveness in minimally invasive HVS.

## GRAPHICAL ABSTRACT

**Figure:**
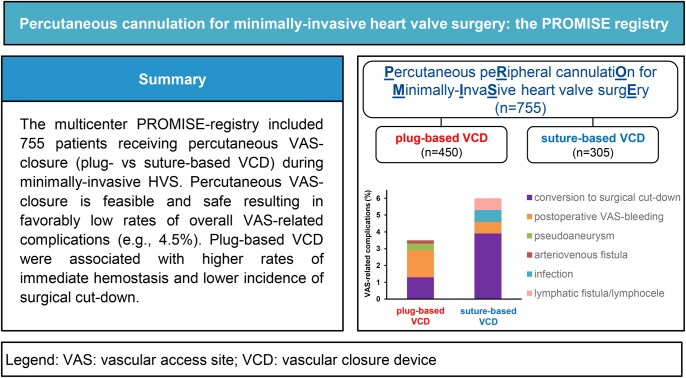

## INTRODUCTION

Heart valve surgery (HVS) via full sternotomy, including central cannulation for cardiopulmonary bypass (CPB), has been gold-standard for the treatment of valvular disease over decades and is supported by current guidelines.^1^ Minimally invasive and endoscopic techniques evolved as alternatives aiming at reduced surgical trauma, less blood transfusions, shorter postoperative ventilation times, and in-hospital stay at specialized centres.^2–5^ Most minimally invasive techniques include groin incision, surgical exposure, and visualization as well as direct cannulation of femoral vessels to establish CPB.^6^^,^^7^ To avoid potential groin incision associated complications (ie, infection, lymphatic fistula, and lymphocele formation), hospital re-admission and further streamline surgery, percutaneous femoral cannulation emerged as an alternative.^8–11^ Therefore, percutaneous vascular access site (VAS) closure is necessary and can be obtained by dedicated vascular closure devices (VCDs), which are routinely used for transcatheter aortic valve implantation (TAVI), as well as endovascular aneurysm repair (EVAR) procedures.^12–19^ VCD can be categorized according to their mechanism: plug- (ie, MANTA, Teleflex, Wayne, PA, United States) vs suture-based (ie, Proglide/Prostyle, Abbott Laboratories, Chicago, IL, United States) techniques. The plug-based technique includes an intraluminal resorbable polymer toggle and an extraluminal haemostatic collagen plug, which are tightened by a polyester suture after arterial decannulation sealing the VAS.^19^^,^^20^ By contrast, suture-based pre-closure technique uses a monofilament polypropylene suture, which is percutaneously deployed via Seldinger technique prior to arterial cannulation, which seals the arterial VAS from the outside after decannulation and is tightly knotted on top of the artery.^21^^,^^22^ Both types of VCD are currently used in clinical practice, although advantages and disadvantages of plug- vs suture-based VCD remain to be investigated.^12–17^^,^^23^^,^^24^

## METHODS

### Ethical statement

The PROMISE registry was designed to assess safety and efficacy of plug- vs suture-based VCD, according to modified Valve Academic Research Consortium (VARC)-3 criteria. It was established in accordance with the Declaration of Helsinki (1964) and approved by the Ethics Committee of the Hamburg Medical Association (# 2023-300341-WF) as well as the ethics committees responsible at each centre. Due to the retrospective, anonymous study design, written informed patient consent was waived. The registry was registered at www.clinicaltrials.gov, Identifier NCT05961150.

### Patients

#### Inclusion criteria

Patients undergoing percutaneous VAS closure using dedicated plug- or suture-based VCD during minimally invasive HVS between 2020 and 2023 at 6 high-volume heart valve centres in Germany were retrospectively included. Retrospective data collection from medical records was obtained using a uniform dedicated, anonymous data base. Indication for surgery was obtained according to the current ESC/EACTS guidelines for the management of valvular heart disease.^1^ Furthermore, decision for a minimally invasive surgical approach was based on local, interdisciplinary Heart Team discussion and standard of care at each participating centre.

#### Exclusion criteria

Patients undergoing full sternotomy and central cannulation, or axillary cannulation for CPB without the use of percutaneous VCD were excluded.

### Surgical setup

All procedures were performed under general anaesthesia by a dedicated team of cardiac surgeons, anaesthesiologists, nurses, and perfusionists according to institutional standards at each participating centre. Transoesophageal echocardiography (TOE) was used to approve indication for surgery. Preoperative computed tomographic (CT) scan to assess the anatomy and potential atherosclerotic calcifications of the aorta and the femoral vessels was not routinely performed. CPB was percutaneously established using femoro-femoral perfusion. Therefore, percutaneous venous and arterial cannulation was performed via Seldinger technique under TOE-guidance using 21-27 French (F) venous and 17-23 F arterial cannulas. After infra-inguinal needle puncture of the mid common femoral artery, heparinization, and insertion of a guidewire, the use of either a plug- or a suture-based VCD was prepared according to institutional standards. To prepare the use of the plug-based MANTA device, the distance between the arteriotomy and the level of the skin was assessed using a dedicated MANTA puncture location dilator prior to arterial cannulation.^19^ For the suture-based technique, a single Proglide device was deployed and the suture was secured prior to insertion of the arterial cannula.^25^ To improve venous drainage, occasionally a second venous cannula was necessary either via the contralateral femoral vein or jugular vein. After weaning from CPB, the femoral venous cannula was removed prior to 1:1 protamine administration. A single mattress stitch was placed above the venous cannulation site followed by manual compression. After adequate venous hemostasis and re-transfusion of remaining CPB blood volume, the tubing of the arterial cannula was punctured, a guidewire was inserted in the femoral artery and visualized in the descending aorta via TOE prior to the removal of the cannula. Using a plug-based VCD, the MANTA sheath was inserted into the femoral artery over a guidewire. After assembly of the device, it was withdrawn until the predetermined deployment level (initially measured distance between arteriotomy and skin and one additional centimeter). After release of the intraluminal toggle, an extraluminal collagen-plug seals the arteriotomy from outside and is tightened prior to cutting the suture and removal of the guidewire. For the suture-based technique, the prepared Proglide knot was tightened on top of the artery according to the manufacturer’s recommendations. In the case of inadequate hemostasis, a second VCD, predominantly an 8F plug-based Angio-Seal (Terumo, Tokyo, Japan) was used prior to removal of the guide wire. A pressure bandage is applied according to institutional standards. Surgical procedures were performed following the local standard of care of each participating centre.

### Study end-points

Primary study end-points were major and minor VAS-related complications according to modified VARC-3 criteria.^26^ Therefore, retrograde dissection, vascular injury (ie, formation of pseudoaneurysm, AV fistula), intraoperative conversion to surgical cut-down, and perioperative vascular intervention rates were analysed. Secondary, immediate hemostasis, the necessity of an additional VCD, the rates of postoperative groin infection, and lymphocele formation, as well as 30-day mortality rates were analysed.

### Statistical analysis

Baseline, perioperative, and FU variables were retrospectively collected in an anonymized, standardized database. Continuous variables are shown as medians (25th percentile, 75th percentile) and compared using the Mann-Whitney *U*-test. Binary variables are shown as counts (frequencies) and compared using the *χ*^2^ test. A *P*-value of <.05 was considered statistically significant. All analyses were performed with R statistical software version 4.0.3 (R Foundation for Statistical Computing, Vienna, Austria).

## RESULTS

### Study population and intraoperative characteristics

**Figure 1.**
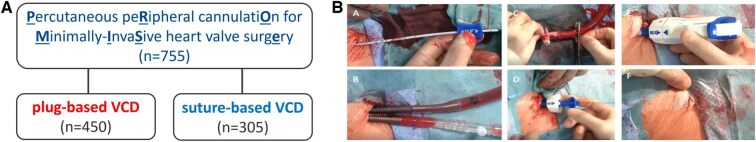
Study Design and Percutaneous Cannulation of Femoral Vessels. (A) Study Design of the PROMISE Registry. (B) Percutaneous Cannulation and VAS Closure Using the Plug-Based MANTA VCD. (A) Assessment of the Distance Between the Arteriotomy and the Level of the Skin, Using a Dedicated MANTA Puncture Location Dilator Prior to Arterial Cannulation. (B) Ready for CPB After Percutaneous Femoral Cannulation with 25F Venous and 21F Arterial Cannulas. (C) Puncturing of Arterial Cannula Tube and Guidewire Insertion Prior to Decannulation. (D) Insertion of the 18F Plug-Based MANTA Sheath Over the Guidewire After Arterial Cannula is Removed. (E) Assembly of the MANTA Device Prior to Withdrawal and Deployment of the Intraluminal Toggle at Predetermined Level. (F) Immediate Hemostasis of VAS After Deployment and Removal of the MANTA Device and Guidewire

Between 2020 and 2023, a total of 755 patients underwent minimally invasive HVS including percutaneous femoral cannulation for CPB and VAS closure using either a plug- (group 1; *n* = 450) or a suture-based VCD (group 2; *n* = 305) at 6 different sites (University Medical Center Hamburg-Eppendorf, University Hospital Bonn, German Heart Center of the Charité, University Hospital Mainz, Leipzig Heart Center and Heart and Diabetes Center North Rhine-Westphalia) in Germany (Figure 1). Baseline characteristics including age, gender, comorbidities, and surgical risk scores are present in Table 1. About 66.1% of patients were male with a median age of 61.9 (54.0-69.0) years. Most prevalent comorbidities were hypertension (53.8%) and atrial fibrillation (AFib) (29.4%), resulting in a median STS Prom score of 0.5 (0.3-0.9)%. Of note, patients showed a median body mass index (BMI) of 24.9 (22.5-28.3) kg/m^2^ and the prevalence of peripheral artery disease was 4.4%. Preoperative CT-assessment of femoral vessels was performed in 35.9% of cases. Indication for surgery was either significant mitral, aortic, or tricuspid valve disease, as well as combinations of those, according to current guidelines.

**Table 1.** Preoperative Patient Characteristics

Variables	All patients (*n* = 755)	Group 1 Plug-based (*n* = 450)	Group 2 Suture-based (*n* = 305)	*P*-value
Age, years, median (IQR)	61.9 (54.0-69.0)	61.0 (54.0-68.0)	62.7 (53.1-72.0)	.019
Male, *n* (%)	499 (66.1)	297 (66.0)	202 (66.2)	>.99
BMI, kg/m^2^, median (IQR)	24.9 (22.5-28.3)	24.8 (22.7-28.4)	25.0 (22.3-27.8)	.74
Diabetes, *n* (%)	66 (8.7)	41 (9.1)	25 (8.2)	.76
Peripheral artery disease, *n* (%)	33 (4.4)	17 (3.8)	16 (5.2)	.43
Arterial hypertension, *n* (%)	335 (53.8)	157 (49.2)	178 (58.6)	.024
COPD > GOLD class I, *n* (%)	38 (5.7)	15 (3.4)	23 (10.3)	<.001
Atrial fibrillation, *n* (%)	222 (29.4)	118 (26.2)	104 (34.1)	.024
Previous stroke, *n* (%)	44 (6.6)	23 (5.3)	21 (9.4)	.059
Coronary artery disease, *n* (%)	144 (21.6)	92 (20.8)	52 (23.3)	.51
NYHA class I, *n* (%)	123 (16.8)	74 (16.7)	49 (16.9)	>.99
NYHA class II, *n* (%)	303 (41.3)	181 (40.8)	122 (42.1)	.78
NYHA class III, *n* (%)	286 (39.0)	173 (39.0)	113 (39.0)	>.99
NYHA class IV, *n* (%)	22 (3.0)	16 (3.6)	6 (2.1)	.33
LVEF, %, median (IQR)	60.0 (55.0-63.0)	60.0 (55.0-63.0)	60.0 (55.0-64.0)	.49
TAPSE, mm, median (IQR)	24.0 (20.0-28.0)	25.0 (20.0-28.0)	21.0 (18.7-25.0)	<.001

Abbreviations: BMI = body mass index; COPD = chronic obstructive pulmonary disease; GOLD = Global Initiative for Chronic Obstructive Lung Disease; IQR = interquartile range; LVEF = left ventricular ejection fraction; NYHA = New York Heart Association; TAPSE = tricuspid annular plane systolic excursion.

Surgical details and perioperative results are summarized in Table 2. Minimally invasive HVS including percutaneous cannulation for CPB was performed in all patients. Distribution of VCD (plug- vs suture-based) according to each participating centre is listed in Table S1. Briefly, mitral valve repair/replacement was performed in 78.5%, aortic valve repair/replacement in 18.8%, and tricuspid valve repair/replacement in 11.1%. Concomitant AFib ablation was performed in 18.7% and occlusion of left atrial appendage (LAA) in 14.6%. Procedural, CPB, and aortic cross-clamp times are listed in Table 2. Rate of sonography-guided arterial puncture using comparably sized femoral artery cannulas (19 (18-19) vs 18 (18-19) F; *P* = .095) was less frequently performed in group 1 (33.6% vs 63.6%; *P* < .001). The prevalence of immediate arterial hemostasis was significantly higher in group 1 (99.8% vs 77.7%; *p* < .001). Use of a second VCD was necessary in 34.8% in group 2 as well as intraoperative conversion rates to surgical cut-down (1.3 vs 3.9; *P* = 0.04) were significantly lower within group 1 (Table 3).

**Table 2.** Intraprocedural Characteristics

Variables	All patients (*n* = 755)	Group 1 Plug-based (*n* = 450)	Group 2 Suture-based (*n* = 305)
Conversion to full-sternotomy, *n* (%)	19 (2.5)	10 (2.2)	9 (3.0)
Endocarditis, *n* (%)	33 (4.3)	22 (4.9)	11 (3.6)
Previous cardiac surgery, *n* (%)	26 (3.4)	4 (0.9)	22 (7.2)
Mitral valve repair/replacement, *n* (%)	593 (78.5)	313 (69.6)	280(91.2)
Aortic valve repair/replacement, *n* (%)	142 (18.8)	135 (30.0)	7 (2.3)
Tricuspid valve repair/replacement, *n* (%)	84 (11.1)	49 (10.9)	35 (11.4)
Atrial fibrillation ablation, *n* (%)	141 (18.7)	72 (16.0)	69 (22.6)
LAA occlusion, *n* (%)	110 (14.6)	91 (20.2)	19 (6.2)
Procedure time, min, median (IQR)	199 (166- 240)	194 (164-233)	205 (167-255)
Cardiopulmonary bypass time, min, median (IQR)	136 (107-176)	134 (102-170)	143 (113-186)
Cross-clamp time, min, median (IQR)	84 (63-109)	80 (61-103)	95 (67-130)

Abbreviations: IQR = interquartile range; LAA = left atrial appendage.

**Table 3.** Cannulation Strategy and Periprocedural Vascular Access Site Related Outcome

Variables	All patients (*n* = 755)	Group 1 Plug-based (*n* = 450)	Group 2 Suture-based (*n* = 305)	*P*-value
Preoperative CT-assessment of femoral vessels and aorta, *n* (%)	271 (35.9)	168 (37.3)	103 (33.9)	.37
CFA diameter, mm, median (IQR)	9.0 (9.0-10.0)	9.0 (9.0-10.0)	10.0 (9.0-11.0)	<.001
Sonography guided arterial puncture, *n* (%)	345 (45.7)	151 (33.6)	194 (63.6)	<.001
Arterial cannula size, *F*, median (IQR)	19.0 (18.0-19.0)	19 (18-19)	18 (18-19)	.095
Immediate hemostasis, *n* (%)	682 (90.3)	445 (98.9)	237 (77.7)	<.001
Deployment of second VCD, *n* (%)	106 (14.0)	0 (0.0)	106 (34.8)	<.001
Conversion to surgical cut-down, *n* (%)	18 (2.4)	6 (1.3)	12 (3.9)	.040
CFA occlusion, *n* (%)	9 (1.2)	4 (0.9)	5 (1.6)	.55
Retrograde dissection, *n* (%)	0 (0)	0 (0)	0 (0)	>.99
Arteriovenous fistula, *n* (%)	1 (0.1)	1 (0.2)	0 (0)	>.99
Femoral pseudoaneurysm, *n* (%)	2 (0.3)	2 (0.4)	0 (0)	.66
Lymphatic fistula/lymphocele formation, *n* (%)	2 (0.3)	0 (0)	2 (0.7)	.32
Groin infection, *n* (%)	2 (0.3)	0 (0)	2 (0.7)	.32
Postoperative VAS-related bleeding, *n* (%)	9 (1.2)	7 (1.6)	2 (0.7)	.26
Any postoperative VAS-reintervention, *n* (%)	15 (2.0)	10 (2.2)	5 (1.6)	.77
Any VAS-related complication, *n* (%)	29 (3.8)	13 (2.9)	16 (5.2)	.14

Abbreviations: CFA = common femoral artery; F = French; IQR = interquartile range; VAS = vascular access site; VCD = vascular closure device.

### VAS-related complications

**Figure 2.**
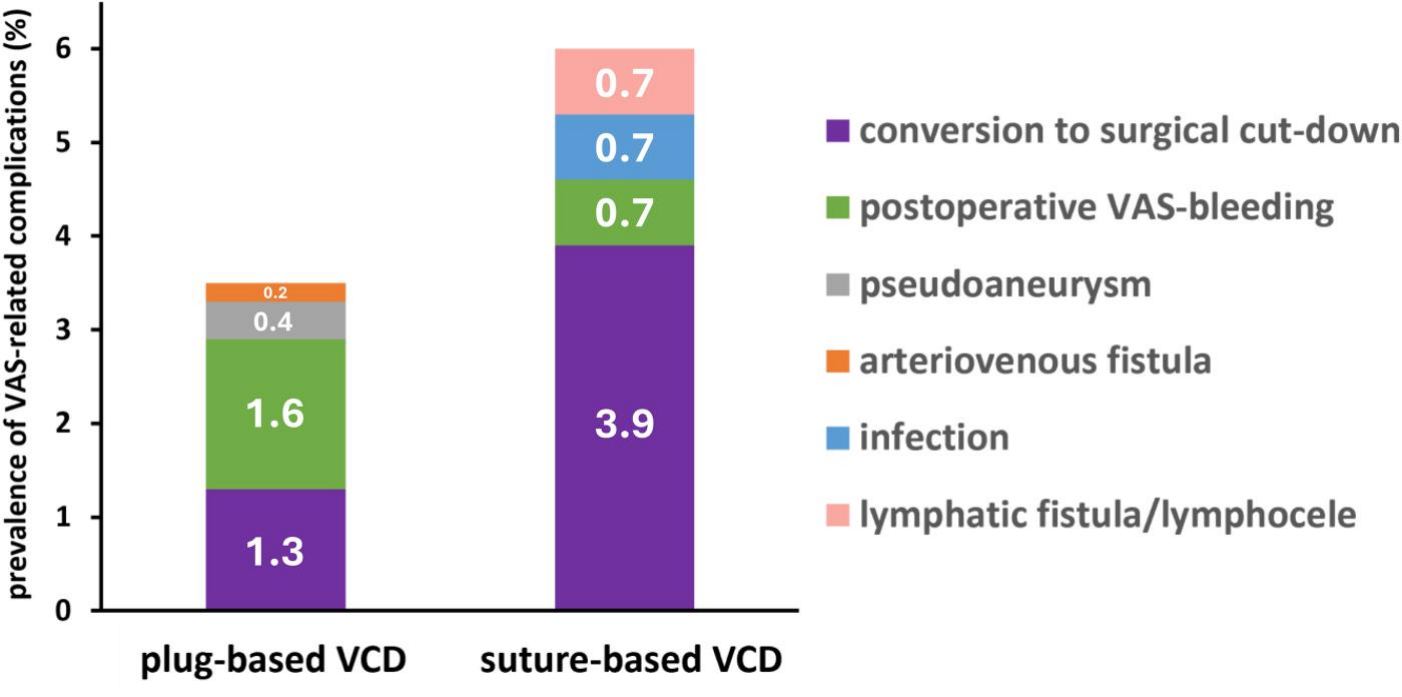
Prevalence of VAS-Related Complications

The overall prevalence of VAS-related complications, which are summarized in detail in Table 3, was favourably low in both the groups (2.9% vs 5.2%; *P* = .14). Of note, there were no cases of retrograde aortic dissection or aortic rupture within both the groups. Furthermore, there were no major VAS-related complications resulting in death, permanent neurologic deficit, or limb amputation. Rates of postoperative lymphatic fistula/lymphocele formation (0% vs 0.7%; *P* = .32), groin infection (0% vs 0.7%; *P* = .32), AV fistula (0.2% vs 0%; *P* > .99), pseudoaneurysm (0.4% vs 0%; *P* = .66), postoperative VAS bleeding (1.6% vs 0.7%; *P* = .26) were similar between the groups (Figure 2).

### Perioperative outcomes and 30-day survival

Perioperative outcome is present in Table 4. Briefly, the necessity of perioperative mechanical circulatory support was 1.5%. The prevalence of re-thoracotomy due to bleeding was 7.9%. In 5.4% of patients, implantation of permanent pacemakers was performed. Median postoperative ventilation time was 4.0 (0.0-7.0) h. Median length of ICU stay was 1.0 (1.0-2.0) days and length of hospital stay was 8.0 (7.0-120) days. Thirty-day mortality was 0.8% within the whole study cohort, whereas no statistical difference between both the groups occurred (0.2% vs 1.6%; *P* = .83).

**Table 4.** Periprocedural and In-Hospital Outcome

Variables	All patients (*n* = 755)	Group 1 Plug-based (*n* = 450)	Group 2 Suture-based (*n* = 305)	*P*-value
ECMO support, *n* (%)	9 (1.2)	4 (0.9)	5 (1.6)	.55
Rethoracotomy, *n* (%)	60 (7.9)	42 (9.3)	18 (5.9)	.12
Permanent pacemaker implantation, *n* (%)	36 (5.4)	21 (4.7)	15 (6.7)	.37
Myocardial infarction, *n* (%)	1 (0.2)	0 (0)	1 (0.4)	.73
Postoperative ventilation time, h, median (IQR)	4.0 (0, 7.0)	3.0 (0-5.0)	9.2 (6.4-12.7)	<.001
ICU stay, days, median (IQR)	1.0 (1.0-2.0)	1.0 (1.0-3.0)	1.0 (0-2.0)	<.001
Hospital stay, days, median (IQR)	8.0 (7.0-12.0)	8.0 (6.0-11.0)	9.0 (7.0-14.0)	<.001
30-Day mortality, *n* (%)	6 (0.8)	1 (0.2)	5 (1.6)	.083

Abbreviations: ECMO = extracorporeal membrane oxygenation; ICU = intensive care unit, IQR = interquartile range.

## DISCUSSION

Percutaneous cannulation of femoral vessels to establish CPB decreases invasiveness in minimally invasive HVS and potentially reduces groin incision associated complications and further streamline surgery. Dedicated VCDs for VAS closure, which are currently routinely used in TAVI and EVAR procedures, represent a potential advancement and their use has become standard of care at specialized centres.

The results of the PROMISE registry demonstrate for the first time in a large, multicentre patient cohort safety and feasibility of percutaneous cannulation with use of VCD during minimally invasive HVS with very low rates of VAS-related complications. Potential advantages of plug-based VCD are superior rates of immediate hemostasis and lower incidence of surgical cut-down due to VCD-failure.

### Study population

Despite significant differences in the prevalence of some comorbidities, the prevalence of peripheral artery disease, which increases the risk of VAS-related complications,^27^ was similarly low in both the groups. Furthermore, normal BMI, which potentially simplifies arterial access and closure,^28^ and use of equally sized cannulas in both the groups, indicate homogeneity regarding risk of VAS-related complications between the groups.

### VAS related complications

Overall VAS-related complications were favourably low in both the groups (2.9% vs 5.2%; *P* = .14). Of note, the rates of VAS-related complications and/or VCD-failure vary from 5% up to 40%^15^^,^^20^ after TAVI. In the randomized CHOICE-CLOSURE trial, comparing plug- vs suture-based VCD during TAVI procedures, patients were significantly older (ie, median age of 80.6 years), had a higher prevalence of comorbidities, and an almost 10-fold increased periprocedural risk (median STS Prom 4.1% and EuroSCORE II 4.6%) as compared to this surgical study cohort. In particular, the low prevalence of peripheral artery disease (4.4% vs 7.6% in the CHOICE-CLOSURE trial), as well as larger median femoral artery diameters (9.0 vs 7.8 mm in the CHOICE-CLOSURE trial) most likely have contributed to low rates of VAS-related complications. Furthermore, in case of severe ilio-femoral or aortic calcification, cannulation of the femoral artery was avoided and instead central cannulation or cannulation of the axillary artery^29^ was performed even though these cases were not included in this registry. Prevalence of groin infection (2/755; 0.3%) or lymphocele formation (2/755; 0.3%) was favourably low in this study and all affected patients underwent surgical cut-down due to VCD-failure during the index procedure. These low rates are in accordance with previous findings on percutaneous cannulation including the use of VCD for VAS closure.^10^^,^^11^ Theoretically, incidence of pseudoaneurysm (2/755; 0.3%) or AV fistula (1/755; 0.1%) may be increased after percutaneous cannulation even though rates were extremely low in this analysis. Still sonographic assessment of the canulation site for puncture as well as after vascular closure is mandatory to detect subclinical VCD-failure and needs to be adapted in all cases. To report about the potential influence of sonography-guided arterial puncture, descriptive subgroup analysis of the whole study population independent of the type of VCD was performed. Noteworthy, no statistical difference between patients undergoing percutaneous cannulation with or without sonography-guided arterial puncture was found in the majority of VAS-related complications (ie, conversion to cut-down (*P* = .73), AV fistula (*P* > .99), formation of pseudoaneurysm (*P* = 0.56), dissection (*pP* > 0.99), as well as overall VAS-related complications (*P* = 0.3)) (Table S1). Importantly, significantly increased rates of arterial occlusion (2.2% (9/410) vs 0.0% (0/345); *P* = 0.015), as well as postoperative bleeding (2.0% (8/410) vs 0.3% (1/345); *P* = 0.03) in patients without sonography-guidance, emphasize the importance of sonography-guided arterial puncture during cannulation for minimally invasive HVS.

Furthermore, due to the lack of routine postoperative imaging of the femoral vessels (ie, via duplex sonography), asymptomatic VAS-related complications (ie, formation of pseudoaneurysm, AV fistula or dissection) were potentially not detected, despite clinical examination prior to discharge.

### Plug- vs suture-based VCDs

The choice of a particular type of VCD in the PROMISE registry was at the investigators’ discretion and followed standard of care at participating centres (Table S1). Noteworthy, each of the participating centres mainly used one type of VCD (plug- or suture-based) at their institutions instead of a homogenous distribution on the basis of an individual case-by-case decision. However, parameters potentially affecting success or failure of VCD, that is incidence of peripheral artery disease, female gender, or obesity^28^ were similar between the groups. Assessment of access vessel characteristics such as artery diameter or tortuosity, or presence of calcification was performed in approximately one-third of patients across groups only, representing room for improvement. Although, the risk of VCD-failure due to relevant atherosclerosis and calcification of femoral arteries is likely to be lower in patients undergoing minimally invasive HVS in comparison to TAVI or EVAR-cohorts, preoperative CT-assessment of the VAS is reasonable, particularly for patients >65 years or with increased risk. In accordance with previous results,^12^^,^^23^^,^^24^^,^^27^ incidence of immediate hemostasis was higher in group 1. Also, use of a second VCD, which is technically possible only when using a suture-based VCD, as well as the rate of conversion to surgical cut-down were significantly increased in group 2. It is important to acknowledge, that particularly in interventional practice (ie, TAVI), it is well recognized that a “two-device” VCD strategy is routinely planned in advance. As such, the deployment of a second VCD in patients treated with suture-based VCD may not represent a device failure, but rather a technical nuance of the VAS closure strategy. Infection and lymphocele formation occurred only in group 2 and only after conversion to surgical cut-down. In addition to differing clinical outcomes, the choice of VCD (plug- vs suture-based) and the associated device strategy (“one—vs two device”) might be associated with potential cost implications. Apart from important differences in device pricing of plug- (ie, MANTA, Teleflex, Wayne, PA, United States) vs suture-based (ie, Proglide/Prostyle, Abbott Laboratories, Chicago, IL, United States) VCD, potentially increased costs due to prolonged procedural time resulting from surgical cut-down, the use of a second VCD and subsequent treatment of VAS-related complications, need to be acknowledged.

## CONCLUSION

Both types of VCD are feasible and safe for VAS closure after percutaneous arterial cannulation to establish CPB during minimally invasive HVS. Advantages of plug-based VCD potentially include higher rates of immediate hemostasis and lower rates of conversion to surgical cut-down. Possible risk factors for VCD-failure such as peripheral artery disease are not as prevalent in patients receiving minimally invasive HVS compared to the general TAVI population. Therefore, differences between these types of VCD in the TAVI literature may not be valid in this surgical patient cohort.

### Study limitations

The present analysis builds on a non-randomized, retrospective study design. Therefore, selection bias as well as confounding factors due to differing institutional treatment standards cannot be excluded and may affect clinical outcomes. Furthermore, the choice of a particular type of VCD in the PROMISE registry followed standard of care at participating centres (Table S1) and is therefore not homogenously distributed on the basis of an individual case-by-case decision. Thus, generalizability as well as drawing conclusions from the results regarding the use of differing VCD is limited. As sample size was not pre-specified, statistical power might be limited, although it represents one of the largest study-cohorts regarding VCD in minimally invasive HVS. Differing baseline characteristics between the study groups may have biased outcomes. However, the incidence of parameters likely affecting VAS closure were similar between the groups. Therefore propensity score matching was not performed. Despite clear advantages, rates of sonography-guided arterial puncture were low within the registry and differed between the groups, potentially influencing clinical outcome. As routine postoperative imaging of the femoral vessels was not performed, asymptomatic VAS-related complications were potentially not detected, nor reported, and VAS-related complication rates might be underestimated. Furthermore, potentially differing postoperative anticoagulation strategies at participating centres might have influenced outcome, particularly regarding perioperative VAS bleeding.

## Supplementary Material

ezaf219_Supplementary_Data

## Data Availability

The data underlying this article will be shared on reasonable request to the corresponding author.
