# Supplementary material for: Percutaneous Cannulation for Minimally Invasive Heart Valve Surgery: Results from a Multicenter Registry
Source: Eur J Cardiothorac Surg. 2025 Jul 2;67(7):ezaf219. doi: 10.1093/ejcts/ezaf219 (PMC12286699; doi:10.1093/ejcts/ezaf219)
Supplement: ezaf219_Supplementary_Data [file ezaf219_supplementary_data.zip › Supplementary S1 and S2.docx]

**Supplementary material**

**Supplementary Table S1: Device distribution within participating centers**

| **Variables** | **All patients**  **(n=755)** | **Group 1**  **plug-based**  **(n=450)** | **Group 2**  **suture-based**  **(n=305)** |
| --- | --- | --- | --- |
| Center 1 | 306 (40.5) | 306 (68.0) | 0 (0) |
| Center 2 | 131 (17.4) | 131 (29.1) | 0 (0) |
| Center 3 | 114 (15.1) | 6 (1.3) | 108 (35.4) |
| Center 4 | 97 (12.8) | 0 (0) | 97 (31.8) |
| Center 5 | 89 (11.8) | 7 (1.6) | 82 (26.9) |
| Center 6 | 18 (2.4) | 0 (0) | 18 (5.9) |

**Supplementary Table S2: Descriptive subgroup analysis according to performance of sonography-guided arterial puncture**

| **Variables** | **All patients**  **(n=755)** | **Group A**  **Not Sono-guided**  **(n=410)** | **Group B**  **Sono-guided**  **(n=345)** | **p-value** |
| --- | --- | --- | --- | --- |
| Immediate hemostasis, n (%) | 682 (90.3) | 395 (96.3) | 287 (83.2) | <0.001 |
| Deployment of second VCD, n (%) | 106 (14.0) | 37 (9.0) | 69 (20.0) | <0.001 |
| Conversion to cut-down, n (%) | 18 (2.4) | 11 (2.7) | 7 (2.0) | 0.73 |
| CFA occlusion, n (%) | 9 (1.2) | 9 (2.2) | 0 (0) | 0.015 |
| Retrograde dissection, n (%) | 0 (0) | 0 (0) | 0 (0) | >0.99 |
| Arteriovenous fistula, n (%) | 1 (0.1) | 1 (0.2) | 0 | >0.99 |
| Femoral pseudoaneurysm, n (%) | 2 (0.3) | 2 (0.5) | 0 | 0.56 |
| Lymphocele formation, n (%) | 2 (0.3) | 0 (0) | 2 (0.6) | 0.40 |
| Groin infection, (%) | 2 (0.3) | 1 (0.2) | 1 (0.3) | >0.99 |
| Postop. VAS-related bleeding, n (%) | 9 (1.2) | 8 (2.0) | 1 (0.3) | 0.03 |
| Any postoperative VAS-re-intervention, n (%) | 15 (2.0) | 12 (2.9) | 3 (0.9) | 0.079 |
| Any VAS-related complication, n (%) | 29 (3.8) | 19 (4.6) | 10 (2.9) | 0.30 |
